# The Possibility of Using Oil Pomace as a Substitute for Walnuts (*Juglans regia* L.) in Muesli Bar Technology

**DOI:** 10.3390/foods13233807

**Published:** 2024-11-26

**Authors:** Patryk Siczek, Justyna Libera

**Affiliations:** 1 Department of Plant Food Technology and Gastronomy, University of Life Sciences in Lublin, Skromna 8 Street, 20-704 Lublin, Poland; patryk.siczek@up.lublin.pl; 2 Department Engineering and Cereal Technology, University of Life Sciences in Lublin, Skromna 8 Street, 20-704 Lublin, Poland

**Keywords:** walnut, muesli bar, by-product, oil pomace, functional food

## Abstract

The utilization of food industry waste to create innovative, high-quality products with reduced environmental impact is a growing trend in food technology. Walnut oil pomace, a byproduct of walnut oil production, is rich in nutrients and bioactive compounds, making it an excellent candidate for reuse in muesli bars as a replacement for walnuts. The aim of the study was to evaluate the possibility of replacing walnuts with oil pomace in muesli bar recipes and to assess whether the resulting product meets quality standards. Ground expeller walnut oil pomaces and aqueous extract were tested, with a bar containing ground walnuts serving as the reference sample. The bars were evaluated for sensory, physicochemical, and antioxidant properties, and their nutritional values were assessed. Results showed that the pomace-enriched bars exhibited satisfactory physicochemical properties, with texture, color, and safety (as measured by water activity and pH) comparable to the control bars. Sensory evaluations classified all bars as acceptable, with no significant differences in nutritional value. The study concludes that walnut pomace holds promise as a sustainable ingredient in food technology, potentially expanding product diversity while reducing environmental impact. This concept aligns with promoting sustainable practices in food manufacturing.

## 1. Introduction

The walnut (*Juglans regia* L.) is valued for its taste, nutritional properties, and medicinal applications [1]. The beneficial effect on the body is determined by the presence of biologically active compounds in nuts, i.e., unsaturated fatty acids, dietary fiber, polyphenols and numerous minerals (zinc, magnesium, calcium) [2,3]. Green fruits, peels, leaves and bark are characterized by a high content of phenolic compounds, while the seeds are rich in essential fatty acids [4,5]. The pith and shell of green walnuts and green leaves are used to make tinctures, teas and infusions, and the leaves can be used for pickling vegetables [6]. Edible walnut seeds are used in the food industry as a raw material for oil extraction. In walnuts, the lipid profile is mostly dominated by PUFAs, with linoleic and linolenic acids being the greatest contributors [4]. Walnut cultivation and consumption continues to increase around the world [5,6,7], with the production of approximately 2.7 million tons in 2023 [8]. At the industrial level, walnuts are widely used as a source of desirable fatty acids, as an ingredient that enhances flavor and aroma [6]. Therefore, walnuts can be used in a wide range of food products, e.g., in bars, snacks, breakfast cereals, and energy bars, in the form of peanut butter, and as a dairy alternative.

The global food industry generates a substantial quantity of by-products annually, many of which remain unused, presenting a significant disposal issue [9]. These include, among others, soybean okra, fruit pomace, bran or pomace, including walnuts [10,11]. The latter are waste after oil extraction from seeds and, due to their high nutritional value, are used as feed, most often for ruminants and fish [12]. The walnut oil extraction industry itself is considered a significant source of waste, the amount of which depends on the pretreatment technology and oil pressing method used [13]. Precise data on the quantity of pomace produced per unit of walnut oil extracted are not widely available. The conversion of food waste into valuable products is referred to as the upcycling process [14]. By-products of the food industry can be used as ingredients for functional foods, which expands the range of products and at the same time reduces the burden on the environment [15]. It is not always possible to reduce the number of by-products, so they should be treated as valuable additional resources for production [16].

Walnuts and their waste products contain a large number of polyphenols, polysaccharides, and other biologically active substances, which have strong development potential for their added value [17]. Nuts have proven beneficial effects on humans, and in combination with a balanced diet they can significantly improve health [2,17,18,19]. Modern lifestyles have led to changes in dietary habits and, consequently, to an increase in the consumption of foods rich in saturated fats, sugars and salt, which contribute to the development of obesity and cardiovascular diseases. Walnut pomace has the potential to be used as an additive in dietary products or as an ingredient in functional foods. A result of milling this pomace is so-called walnut flour, which is a good source of protein and carbohydrates [10]. Including products containing walnut pomace in your daily diet can help improve your body’s condition due to the richness of antioxidants [20,21]. Polyphenols in walnuts have antioxidant, anti-inflammatory, anti-tumor, and cardiovascular-protective abilities [22]. The ellagic acid content (ca. 0.5–1.7 mg g^−1^) in pomaces from walnut *Diaphragma Juglandis* fructus was considerable and comparable to or even higher than that in blackberries, cloudberries, strawberries, raspberries, fruit juices, and walnut kernels [23]. Most phenolic compounds are found in the fatty nut pulp, while these compounds remain in the pomace after cold pressing [24,25]. The use of walnut pomace in the technology of muesli bars can not only improve their nutritional quality, but also have a positive impact on the technological process, e.g., on the correct color or improved water absorption [10,26].

Muesli bars are a popular snack, making it worthwhile to expand the range with innovative variations on classic recipes, especially since consumption forecasts indicate that the global snack bar industry will grow by about USD 4 billion between 2019 and 2025 [27]. This study aimed to evaluate the possibility of substituting walnuts in muesli bars with oil pomace to promote waste reutilization in food technology. This approach seeks to balance the use of by-products from food processing with preserving the high quality and sensory appeal of the modified product. The use of an aqueous extract of oil pomace in muesli bar technology has not yet been described in the literature, making its inclusion in this study an innovative approach.

## 2. Materials and Methods

### 2.1. Materials

All ingredients used in the study were purchased in a retail supermarket in Poland, immediately before the study began. Organic oat flakes (Melvit), multi-flower nectar honey (Apis), and virgin coconut oil (goBIO) were used. Dried cranberries, sunflower seeds, coconut flakes, flax seeds and walnuts were purchased from the Bakalland brand. The walnut oil pomace was obtained from the oil mill of Farmy Roztocza, Księżpol, Poland. The reagents used for analyses were analytical grade.

### 2.2. Preparing of Walnut Oil Pomaces Extract

Aqueous extracts of oil pomace were prepared based on prior, unpublished research results. Exactly 30 g of dried pomace was ground precisely in a grinder Ronic Partner (Ronic Poland Sp. z o.o., Łódź, Poland). Then, 350 mL of hot distilled water was added, and the suspension was shaken and heated for 30 min on a laboratory shaker (Water Bath Shaker Type 357, Elpan, Wrocław, Poland) at 150 rpm and at a water temperature of 85 ± 5 °C in the bath. Then, the suspension was thoroughly separated in an MPW-350R centrifuge (MPW Med. Instruments, Warszawa, Poland) at 5000× *g* for 10 min. The supernatant was filtered through Whatman no. 1 paper, and the obtained volume of the extract (approximately 70 mL) was transferred to a round-bottom flask and concentrated in a vacuum evaporator (Rotavapor® R-215, Büchi®, Flawil, Switzerland) in a water bath at 50–60 °C and 250–300 hPa pressure. After evaporating 50% of the solvent, the final extract was obtained and transferred to 12 plastic Eppendorf containers with a capacity of 2 mL each. The mass of the concentrated extract was 33 g and the volume was 24 mL. On this basis, the density was estimated at 1.375 g/dm^3^. The containers were tightly closed and stored in the freezer until the muesli bars were formulated. Due to its thick consistency, the extract was diluted with water in a 1:1 ratio before application.

### 2.3. Formulation of Muesli Bars

Oat flakes and dried cranberries were ground in a grinder (Ronic Partner, Ronic Poland Sp. z o.o., Łódź, Poland) for 5 s at the device’s maximum power. Next, the remaining dry ingredients (linseed and sunflower seeds) were added and gently mixed with a spatula. Three mixture variants were prepared: with the addition of Ground Walnut oil Pomace (GWP variant), an aqueous Extract of Walnut oil Pomace (EWP variant), or ground walnuts (control variant, CON). The added amounts were 9.5% ground walnut or ground oil walnut pomace or 0.3% extract. Based on prior calculations, it was determined that 3 g of extract corresponded to 9.5 g of ground pomace. Walnuts and pomace were ground in a grinder (Ronic Partner, Ronic Partner Sp. z o.o., Łódź, Poland) for 5 s at maximum power immediately before being added to the mixture. The remaining ingredients (coconut oil and honey) were liquefied in a water bath and then combined with the dry ingredients. Each mixture was kneaded by hand for 60 s until a homogeneous mass was achieved. Rectangular bars, each weighing 30 g, were then formed, packaged in string bags, and refrigerated at 4 °C for 5 h before undergoing analysis. The recipes are presented in Table 1.

The extract from the muesli bars was analyzed to determine pH, total phenolic content (TPC), and DPPH assays. It was prepared as follows: the mixture was obtained by homogenizing muesli bars with distilled water in a ratio of 1:9 for 60 s using an MQ5025 Multiquick 5 Vario hand blender (Braun Household GmbH, Neu-Isenburg, Germany). The suspension was then thoroughly separated in an MPW-350R centrifuge (MPW Med. Instruments, Warszawa, Poland) at 5000× *g* for 10 min. The supernatant was filtered through Whatman no. 1 paper into Falcon plastic containers and stored in the refrigerator until the muesli bars were analyzed.

### 2.4. Physicochemical Characteristic of Muesli Bars

The water activity (a_w_) of muesli bars was determined in triplicate using an a_w_-analyzer (LabMaster Novasina AG, Lachen, Switzerland) at 25 °C, according to the manufacturer’s instructions.

The pH measurement was carried out using a CPC-501 digital pH-meter (Elmetron, Zabrze, Poland) equipped with a pH ERH-111 electrode (Hydromet, Bytom, Poland) at 25 °C, according to the manufacturer’s instructions.

The color of the muesli bars were measured using a Konica Minolta CR-5 chroma meter (Konica Minolta Business Solution Europe GmbH, Langenhagen, Germany) applying the CIELAB scale (L*, a*, and b*). Color measurements were performed at room temperature at six different locations on the bars. The conditions were 13 mm port size, illuminant D65 and 10° standard observer. The Konica Minolta’s white and black standards were used to calibrate the spectrophotometer. The total color difference (ΔE*) was calculated based on ΔL*, Δa* and Δb* results for each sample according to the control variant, as follows: ΔE* = [(ΔL*)^2^ + (Δa*)^2^ + (Δb*)^2^]^1/2^.

Texture profile analysis (TPA) of the muesli bars was determined in 15 replications using a Texture Analyzer (TA.XT2, Stable Micro Systems, Godalming, UK) equipped with the P/75 attachment and the HDP/90 stage. A double compression cycle test was performed at up to 50% compression of the original portion height with a cylinder probe. A time of 5 s was allowed to elapse between the two compression cycles. Force-time deformation curves were obtained with a 50 kg load cell applied at a cross-head speed of 5 mm s^−1^. The TPA test parameters were quantified as hardness, springiness, cohesiveness, gumminess and chewiness. Calculations of the texture parameters were performed using standard computational methodologies implemented in the device control software (Texture Exponent v32).

### 2.5. Antioxidant Properties of Muesli Bars

Total phenolic compounds (TPC) in the extract of muesli bars were determined using the Folin–Ciocalteau method according to Singleton et al. [28]. The results showed the value of gallic acid equivalent (mg GAE mL^−1^) and were determined from the following regression equation based on the established calibration curve (y = 3.6373x + 0.0994; R^2^ = 0.9872, where y is the absorbance and x is the gallic acid concentration).

The antioxidant activity as DPPH radical scavenging activity was determined spectrophotometrically (at 517 nm) using 2,2-diphenylpicrylhy-drazyl (DPPH) radicals. Refer to the method of Brand-Williams et al. [29] The DPPH radical scavenging activity was determined using the formula:

DPPH radical scavenging (%) = 1 − [(Abs_control_ − Abs_sample_)/Abs_control_] × 100%

### 2.6. Sensory Evaluation of Muesli Bars

For the sensory evaluation of muesli bars, the Quantitative Descriptive Profile (QDP) described by Szydłowska et al. [30] was used with a slight modification by the authors. The study was conducted in accordance with the Declaration of Helsinki, and the protocol was approved by the Ethics Committee for no. KE/38/2024 (Keep). The freshly manufactured samples were assessed by 23 panelists (students and employees) of the Faculty of Food Science and Biotechnology of the University of Life Sciences in Lublin. Before evaluation, the panelists were trained in the methodology. The authors prepared descriptors for the sensory test (Table 2). Bars were divided into 10 g portions and placed in plastic boxes (125 mL) covered with lids and kept at room temperature (22 °C) for 30 min before analysis. Two bars assessed (EWP and GWP) were coded, and the CON was designated as the reference sample. The authors’ modification of the method consisted of the sensory panel assessing the quality of the coded muesli bars based on qualitative features, defining the evaluated feature as “less intense” (−5 ÷ −1) or “more intense” (+1 ÷ +5) compared to the control sample (0 value). The task of the panelist was to determine the intensity of each of the quality features and put their assessment on an unstructured, 100 mm graphic scale (from −5 to +5 contractual units, c.u.).

### 2.7. Proximate Nutritional Value of Muesli Bars

The nutritional value of the muesli bars was estimated based on the recipe composition (the exact amount of raw materials used), using the information included on the labels of the purchased raw materials. The energy value was computed using the Atwater general factor system: carbohydrate (4 kcal g^−1^), lipid (9 kcal g^−1^), and protein (4 kcal g^−1^) [31].

### 2.8. Statistical Analysis

The collected data were analyzed using one-way analysis of variance (ANOVA) to assess the effects of muesli bar formulations on selected parameters. The experiment was conducted in two independent series, with each analysis performed in at least triplicate for accuracy. Statistical calculations were conducted using Microsoft Office Excel 2013 (Microsoft Corporation, Redmond, WA, USA) and Statistica 10 (StatSoft Polska Sp. z o.o., Kraków, Poland). All groups met the assumption of normality (*p* > 0.05). Post hoc analysis was performed using Tukey’s test to identify significant patterns and relationships among subgroups. Statistical significance was set at *p* ≤ 0.05. Results are presented as means ± standard deviation.

## 3. Results and Discussion

Water activity (aw) is an important indicator that characterizes the content of free water necessary for microbial growth, and therefore its values are useful for predicting the microbiological stability of the product. High aw-values (>0.8) pose a microbiological risk to the product because they are optimal for microorganism growth; for mycotoxigenic mold, the lower growth limit has been reported to be 0.78 aw [32]. Therefore, it is important that the product has lower water activity, providing a natural margin of safety. Water activity (Table 3) is an important parameter that determines the quality and safety of food. The aw-values of all the assessed muesli bars were similar (from 0.64 to 0.66). The EWP bar had the highest aw-value, but it was not a statistically significant difference (*p* ≤ 0.05). Similar water activity was found in high-protein muesli bars (0.63–0.69) [30] and bars with date paste (0.65–0.72) [33], while higher aw-values (0.78–0.85) were also reported in articles of this parameter in high-energy protein bars [33].

The next quality parameter of muesli bars, which strictly determines their quality, is the pH value (Table 3). The bars were characterized by a low pH (ranging from 4.57 to 5.43), and the test samples had significantly (*p* ≤ 0.05) lower values compared to the control sample, which proves their microbiological safety. Other researchers found higher values of the pH parameter (6.3–7.0) in high-protein muesli bars [30].

The color (Table 3) of muesli bars is an important parameter determining consumers’ choices. In this study, the CON muesli bar was used as a reference sample, and the colors of the test bars (GWP and EWP) were compared to it. The calculated total color difference (ΔE*) should not exceed 2.5 units, as values above this threshold indicate different coloration. The ΔE* values for the GWP and EWP samples were 1.6 and 1.8, respectively, demonstrating their similarity in color to the reference sample. All muesli bars had identical lightness, expressed as the L* index (average 52.4 ± 0.5 c.u.), and redness (5.1 ± 0.4 c.u.). The yellowness, expressed by the b* index, turned out to be the only difference in colors of the muesli bars. The control sample (17.0 ± 1.4 c.u.) had a significantly lower (*p* ≤ 0.05) value so it was less yellow than the samples: GWP (18.6 ± 1.7 c.u.) and EWP (18.7 ± 1.4 c.u.). The obtained results of instrumental color parameters indicate that the tested bars were brown, typical of muesli/granola bars.

A common issue in snack bar production is the stickiness caused by soluble sugars, which impacts the texture and sensory qualities, resulting in a softer consistency [33]. Texture evaluation with the TPA double compression test showed that all muesli bars had similar hardness, although there were significant standard deviations (25–50% of mean value) during testing (Table 3). This was due to the heterogeneous structure of muesli bars. Plant additives can influence the texture of bars, e.g., dates can be used as an ingredient that retains moisture in the storage of this type of food product [33].

The average hardness of all muesli bars was 224 N ± 85, and variants GWP, EWP and CON did not differ statistically, respectively: 232 N, 189 N and 252 N. Similarly with the chewiness, which was average 273 g ± 149. Differences were found between springiness and cohesiveness in the tested bars. The sample with the extract performed significantly (*p* ≤ 0.05) higher for these values (S: 0.11 ± 0.04, C: 0.16 ± 0.04) compared to the other samples. Both samples of bars with the addition of walnut pomace had greater chewiness than the control sample, especially EWP-bars (*p* ≤ 0.05). The obtained results for cohesiveness are similar to those of Dimopoulou et al. [34], who tested muesli bars for diabetics. On the other side, hardness tests for bars containing *Coprinus comatus* protein powder showed higher values, from 304 to 429 N, so these bars were harder than muesli bars prepared by the authors of this study [34].

Muesli bars contain walnuts in their formulation in various forms, making them a source of antioxidant compounds. The total polyphenol content and the antioxidant activity defined as % DPPH inhibition was assessed (Table 4).

The highest DPPH inhibition value was achieved by the variant that contained walnuts, i.e., the control sample (56.7%), while a slightly lower value was achieved by the variant containing walnut oil pomace (52.8%). The EWP bar had the lowest antioxidant activity value, at 1/3 less than the GWP bar. It can be seen that the DPPH inhibitory capacity decreases with the change in walnut form. The differences obtained between the variants were statistically significant at (*p* ≤ 0.05). Similarly, the results for total polyphenol content (TPC) in bars were ranked. The CON sample with the addition of ground walnuts had the highest value (0.13 mg GAE mL^−1^). The differences obtained between the variants were significant (*p* ≤ 0.05). There are no published research results on muesli bars with walnut pomace to which our antioxidant activity results could be compared. Slightly higher antioxidant activity (61–69% DPPH inhibition) was reported by Szydłowska et al. [30], though it should be noted that their muesli bars were high in protein. Similar DPPH inhibition results (32.3–50.5%) were obtained in another study [35], where the authors attributed the antioxidant activity to the increased phenolic content. Moreover, the TPC activity was higher (0.46 mg GAE g^−1^) in reformulation bars as compared to the control (0.13 mg GAE g^−1^) due to the high content of functional food present in the protein bars [35]. Muesli bars seem to be a good source of antioxidants, especially since the manufacturing process can often be performed without heat treatment.

**Figure 1:**
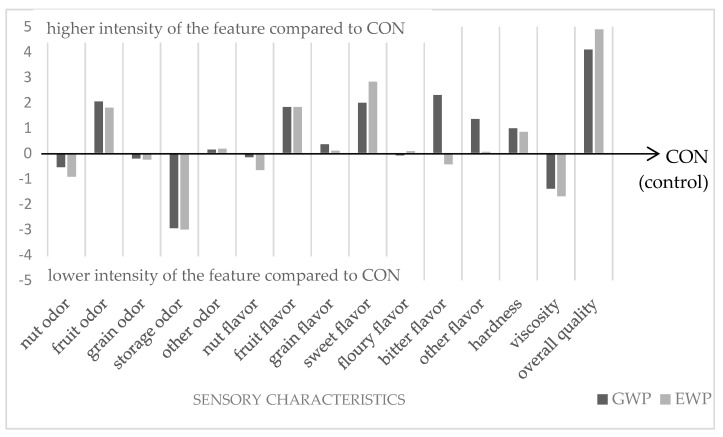
Results of sensory evaluation of fresh muesli bars (QDP method). Explanatory note: a value of zero on the vertical axis indicates the reference sample (CON). Values above mean higher intensity of the feature, and values below mean lower intensity of the feature.

The muesli bars were subjected to sensory evaluation (Figure 1) immediately after formulation and cooling. All three variants were similar to each other in terms of the intensity of the nut and cereal odor and flavor. The panelists assessed both bars and found that GWP and EWP had a slightly sweeter (2.0–2.8 c.u.) taste, and the storage odor was less noticeable (−3.0 c.u.). This is probably related to the lower fat content in these samples. According to the panelists’ evaluation, both variants (GWP and EWP) differed in their bitter flavor and the presence of the other flavor. The GWP muesli bar had a more intense (2.3 c.u.) bitter taste than the others. The GWP and EWP variants had similar levels of fruity odor and flavor, with these features being more intense than in the CON bar. The texture of the bars tested was assessed as harder than in the control sample, and the viscosity as lower. The GWP and EWP variants were highly (above 4 c.u.) assessed in the sensory evaluation, and the EWP bar achieved the highest score (+4.89 c.u.) in the overall quality category. A similar sensory evaluation of muesli bars was conducted by Szydłowska et al. [30], but they used a different scale from 1 to 10. In their study, muesli bars were characterized by the intensity of the cereal and nutty aroma impression at the level of about 5 c.u. The intensity of the taste was noted as follows: cereal; nutty and salty, and the overall quality was noted at the level of 6.8–7.2 c.u. [30]. Ibrahim et al. conducted a sensory evaluation of the bars to determine the optimal sensory characteristics and found that the overall acceptability, appearance, taste, sweetness and texture were perceived without significant differences between the control sample and those supplemented with different concentrations of the date snack bar [33]. Therefore, it seems that different recipes of muesli bars do not negatively affect their quality and sensory characteristics.

The recipe composition and nutritional value of a muesli bar are important because it is often classified as a high-sugar food. If the bar recipe includes a source of protein, fiber, and polyunsaturated fats, and is therefore designed appropriately, it can be considered a functional food [36]. The nutritional value of a food product, defined by the type and quantity of nutrients it contains and their bioavailability, determines its overall usefulness. Nutritional value also serves as an indicator of nutrient density, reflecting the amount of energy released within the body during the biological oxidation of macronutrients [33]. One muesli bar provides 6–7% of the daily energy requirement and can be a valuable snack between meals, but due to the high sugar content (ca. 7 g), these portions should not be exceeded (Table 5). In the present study, muesli bars contained dietary fiber above 3 g per 100 g, and could therefore be considered a food is a source of this ingredient [37]. Such information could be included on the bar label in the form of a nutrition claim, as this would help consumers make informed food choices.

The energy density values of the muesli bars calculated in this study (374–435 kcal) are comparable to the average values of bars available on the Australian market (423 kcal). Additionally, they have similar average protein (8 g) and carbohydrate (57 g) content per 100 g of product [38]. Comparing the obtained results to other studies on protein-rich bars, it can also be concluded that the energy value of 100 g of the product may range from 315 [34] to 390 kcal [30] and this is due to the large share of protein in their recipe composition. However, due to the presence of walnuts and coconut oil in the recipe composition of our bars, we obtained a higher (more by 5–10 g) fat content. The sample muesli bar, containing oil pomace instead of walnuts, has lower energy (374 kcal) and a lower fat content (18.5 g).

## 4. Conclusions

Developing sustainable food sources to meet global nutritional needs is a priority for the food industry, and may involve the industrial and technological utilization of novel food sources. The global food industry produces substantial quantities of by-products annually, many of which remain unutilized, posing significant disposal challenges. The pomace generated as a by-product in the processing of edible oils can be repurposed to create products with properties comparable to conventional foods. The present study demonstrated that pomace derived from walnut oil and its extract can be incorporated into muesli bar technology as a substitute for walnuts. The addition of pomace did not adversely affect the acceptability of the product’s appearance. Physicochemical analyses indicated that substituting walnuts with pomace did not alter the water activity, pH values, or color, or significantly modify the texture of the muesli bars. Sensory evaluation revealed that bars containing pomace received favorable ratings, comparable to those of control bars made with walnuts. These findings underscore the potential of utilizing waste materials in the development of innovative muesli bar formulations. Further investigations are warranted to explore the full potential of pomace in food technology.

## Figures and Tables

**Table 1:** Formulation of muesli bars for research variants.

Ingredients	Variants of Muesli Bars		
	GWP	EWP	CON (Control)
oat flakes	355 g	355 g	355 g
honey	240 g	240 g	240 g
coconut oil	100 g	100 g	100 g
dried cranberries	95 g	95 g	95 g
sunflower seeds	60 g	60 g	60 g
shredded coconut	30 g	30 g	30 g
linseed	25 g	25 g	25 g
ground walnuts	—	—	95 g
ground walnut oil pomace	95 g	—	—
extract of walnut oil pomace	—	3 g	—
distilled water	—	3 g	—
Total mass of mixture	1000 g	908 g	1000 g

Explanatory notes: GWP—Ground Walnut oil Pomaces; EWP—aqueous Extract of Walnut oil Pomace; CON—ground walnuts (control variant).

**Table 2:** Descriptors and the marks of anchors defined in the QDP.

Quality Features	Descriptor	The Marks of Anchors
Odor	intensity of nut odor intensity of fruity odor intensity of grain odor intensity of storage odor intensity of other odor	less intense (−5 c.u.) — the reference sample CON (0 c.u.)—more intense (+5 c.u.)
Flavor	intensity of nut flavor intensity of fruity flavor intensity of grain flavor intensity of sweet flavor intensity of floury flavor intensity of bitter flavor intensity of other flavor	less intense (−5 c.u.) — the reference sample CON (0 c.u.)—more intense (+5 c.u.)
Texture	hardness viscosity	less intense (−5 c.u.) — the reference sample CON (0 c.u.)—more intense (+5 c.u.)
Overall quality		worse—better (than the reference sample CON)

**Table 3:** Physicochemical characteristic of muesli bars.

Parameter		Variants of Muesli Bars		
		GWP	EWP	CON (Control)
Water activity [−]		0.644 ^a^ ± 0.014	0.661 ^a^ ± 0.008	0.657 ^a^ ± 0.002
pH value [−]		5.20 ^b^ ± 0.06	4.57 ^c^ ± 0.05	5.43 ^a^ ± 0.03
Color parameter	L* (lightness)	51.94 ^a^ ± 2.38	52.84 ^a^ ± 2.67	52.29 ^a^ ± 2.04
	a* (redness)	4.99 ^a^ ± 0.74	5.46 ^a^ ± 0.82	4.77 ^a^ ± 0.52
	b* (yellowness)	18.57 ^a^ ± 1.68	18.66 ^a^ ± 1.43	17.05 ^b^ ± 1.44
	ΔE* (total color difference between tested bars and CON)^1^	1.58	1.84	-
Texture profile analysis (TPA)	Hardness [N]	232 ^a^ ± 122	189 ^a^ ± 59	252 ^a^ ± 50
	Springiness [−]	0.08 ^b^ ± 0.01	0.11 ^a^ ± 0.04	0.07 ^b^ ± 0.01
	Cohesiveness [−]	0.12 ^b^ ± 0.05	0.16 ^a^ ± 0.04	0.10 ^b^ ± 0.01
	Gumminess [g]	260 ^a^ ± 191	305 ^a^ ± 161	253 ^a^ ± 74
	Chewiness [g]	23.0 ^ab^ ± 20.1	35.5 ^a^ ± 29.1	17.3 ^b^ ± 6.7

Explanatory notes: ^a–c^ means followed by the same letter in the row do not differ statistically among themselves by the Tukey test (*p* ≤ 0.05). ^1^ ΔE* values below 2.5 are imperceptible.

**Table 4:** Antioxidant properties of muesli bars.

Parameter	Variants of Muesli Bars		
	GWP	EWP	CON (Control)
TPC [mg GAE mL^−1^]	0.11 ^b^ ± 0.01	0.06 ^c^ ± 0.00	0.13 ^a^ ± 0.01
DPPH [% radical scavenging]	52.78 ^b^ ± 0.71	36.03 ^c^ ± 0.65	56.71 ^a^ ± 0.87

Explanatory notes: ^a–c^ means followed by the same letter in the row do not differ statistically among themselves by Tukey test (*p* ≤ 0.05).

**Table 5:** Nutritional value of muesli bars (per 30 and 100 g of product and % daily value).

Parameter	GWP			EWP			CON		
	100 g of Product	Serving Size (30 g)	% of the DV *	100 g of Product	Serving Size (30 g)	% of the DV *	100 g of Product	Serving Size (30 g)	% of the DV *
Energy (kcal)	374	112	6%	389	123	6%	435	131	7%
Protein (g)	6.7	2.0	2%	6.3	1.9	2%	7.8	2.3	3%
Total Fat (g)	18.5	5.6	10%	17.9	5.4	10%	24.2	7.3	13%
- saturated (g)	10.6	3.2	n/a	10.6	3.2	n/a	11.3	3.4	n/a
Carbohydrates (g)	47.9	14.4	5%	53.6	16.1	6%	49.3	14.8	5%
- sugars (g)	21.3	6.4	n/a	22.1	6.7	n/a	22.5	6.8	n/a
- dietary fiber (g)	5.1	1.5	5%	4.4	1.3	4%	5.2	1.6	5%

* Percent Daily Values (DV) are based on a 2000 calorie diet. Explanatory note: n/a—not available.

## Data Availability

The original contributions presented in the study are included in the article, further inquiries can be directed to the corresponding author.
